# CMOS: Efficient Clustered Data Monitoring in Sensor Networks

**DOI:** 10.1155/2013/704957

**Published:** 2013-12-25

**Authors:** Jun-Ki Min

**Affiliations:** School of Computer Science and Engineering, Korea University of Technology and Education, Byeongcheon-myeon, Cheonan, Chungnam 330-708, Republic of Korea

## Abstract

Tiny and smart sensors enable applications that access a network of hundreds or thousands of sensors. Thus, recently, many researchers have paid attention to wireless sensor networks (WSNs). The limitation of energy is critical since most sensors are battery-powered and it is very difficult to replace batteries in cases that sensor networks are utilized outdoors. Data transmission between sensor nodes needs more energy than computation in a sensor node. In order to reduce the energy consumption of sensors, we present an approximate data gathering technique, called CMOS, based on the Kalman filter. The goal of CMOS is to efficiently obtain the sensor readings within a certain error bound. In our approach, spatially close sensors are grouped as a cluster. Since a cluster header generates approximate readings of member nodes, a user query can be answered efficiently using the cluster headers. In addition, we suggest an energy efficient clustering method to distribute the energy consumption of cluster headers. Our simulation results with synthetic data demonstrate the efficiency and accuracy of our proposed technique.

## 1. Introduction

The sensors in a wireless sensor network generate a large amount of data that must be communicated to the base station using radio transmission. In particular, the limitation of power is critical since most sensors are battery-powered and it is very difficult to replace batteries in cases that sensor networks are utilized outdoors. Like related literature [1–3], we consider the minimization of the transmission overhead since it is known that the transmission cost is much higher than sensing cost and computing cost. Many techniques in diverse areas such as the routing protocol [4, 5], event detection [3, 6, 7], in-network aggregation [1], and approximate data gathering [2, 8] have been proposed in order to reduce the communication overhead.

In-network aggregation provides a great opportunity for reducing the communication overhead using summary data (e.g., SUM) and/or exemplary data (e.g., MIN and MAX). However, a single aggregated value is insufficient to analyze the whole sensor field in some applications [9]. In addition, outliers may incur large errors in a single aggregation value.

Since a user may want to collect all sensor readings without any aggregation in order to obtain a data set that will support further off-line analysis, a common mode of sensor networks is gathering and detecting critical events in a physical environment [9]. Furthermore, in a large sensor network, sensor readings may not accurately reflect the current state of the network due to device noise, network failure, and so on. Thus, in many cases, users are interested in individual readings of sensors, rather than aggregated data. For instance, consider a sensor network deployed for habitat monitoring. An objective is to monitor and correlate the sensor readings for trend analysis, detecting outliers, or other adverse events. Therefore, some data gathering techniques [2, 8, 10] in sensor networks have been proposed. Periodic reporting of sensor readings drains the energy of sensors since it results in excessive communication. Thus, to reduce the communication overhead, in-network approximation techniques have been proposed. The in-network approximation exploits the fact that a large number of applications can tolerate approximate sensor readings.

Generally, in approximate techniques, each sensor estimates $\hat{v}$ its reading *v* using a certain prediction model. If the difference of $\hat{v}$ and the actual reading *v* is greater than a user specific error bound *ϵ* (i.e., $|\hat{v}-v|>\epsilon$), each sensor transmits *v* to the base station. In the base station, a mirror model is maintained to predict a sensor reading of each sensor. Thus, if a sensor node does not send a sensor reading, the base station obtains an approximated sensor reading using the mirror model. However, for most techniques of this approach, each sensor estimates its reading independently. A sensor's neighbor refers to any other sensor that is within its communication distance. In the sensor field, the spatial correlations such that the change patterns of two neighbors' sensor readings are the same or similar occur. Therefore, in this paper, we propose CMOS, a cluster based monitoring technique for sensor networks utilizing the spatial correlation. The goal of CMOS is to obtain sensor readings within a certain error bound efficiently. To estimate sensor readings, CMOS utilizes the Kalman filter which requires only the previously predicted future value and the current measure value to predict a future value.

CMOS has the following combination of contributions in order to gather sensor readings in an energy efficient manner.

- Our estimation of sensor readings is based on the spatial correlation such that the change patterns of sensor readings of the neighbor sensors are the same or similar. Following the spatial correlation, although sensor readings of two neighbor sensors change, the difference of two sensor readings is stable (or estimative). In CMOS, the difference of neighbor nodes is estimated by the Kalman filter.
- In order to utilize the spatial correlation, CMOS groups sensors as clusters. In each cluster, there is a cluster header. Each cluster header estimates differences of its own reading and members' readings.
- Since the energy consumption of a cluster header is greater than the other sensors, a sensor should avoid acting as a cluster header permanently. Thus, we devise a simple but robust cluster management technique. The basic idea of our cluster management technique is that a sensor having a great amount of energy will act as a cluster header. Since each sensor makes autonomous decision, our clustering mechanism is robust.
- To demonstrate the efficiency of CMOS, we provide an extensive experimental study of our technique using synthetic data sets and compare our technique with the previous approaches. Experimental results show that our proposed technique reduces the communication overhead compared to the other approaches.

*Organization of this Paper*. In the remainder of this paper, we present the details of CMOS with the following organization.  Section 2 presents various sensor data management techniques. In Section 3, we describe the basics of the Kalman filter. In Section 4, we describe the data model and the mechanism of our proposed techniques.  Section 5 contains the performance study. Finally, in Section 6, we summarize our work.

## 2. Related Work

One of the well-known approaches to reduce the energy consumption of sensor networks is the in-network aggregation. In the in-network aggregation, a traditional approach is that partial aggregated results are progressively merged at intermediate nodes on their way to the base station according to the tree routing [1]. Approximate and robust aggregation techniques have been also proposed. The work of Considine et al. [11] and Nath et al. [12] was based on the sketch theory and multiple path routing. In the work of [13], a compression technique, called *q-digest*, was introduced in order to support not only simple aggregation functions (e.g., SUM and MIN) but also MEDIAN. Recently, Silberstein et al. [14] presented an efficient algorithm for the exemplary aggregation. In addition, the work of [15] considered the minimization of communication by combining the processing of multiple aggregations over a fixed tree routing.

Recently, effective in-network aggregation techniques [16, 17] using the Kalman filter were proposed. In this work, in order to detect the false injected value, the estimated aggregation value is obtained using the Kalman filter. If the gap between the estimated aggregation value and the actual aggregation value is greater than the threshold, the estimated aggregation value is decided as a falsely injected value.

Although aggregation measures are sufficient in many applications, there are situations where they may not be enough. For these situations, some sensor data gathering techniques have been proposed [2, 8, 9, 18].

A simple way to reduce the communication overhead is the *temporal suppression*, in which a node transmits its reading if the reading has changed after the last transmission. This policy keeps nodes from repeatedly sending identical data and is greatly beneficial in a mostly unchanging environment. However, sensor readings generally change over time. When sensor readings change significantly at a sensor, the energy of the sensor is drained in order to send the sensor reading to the base station.

Most applications of sensor networks do not require highly accurate data. Therefore, some approximated data gathering techniques were introduced. Earlier work on processing the data stream proposed the caching of a value interval instead of a value at a sensor and the base station and suggested that a sensor should refrain from propagating its values as long as they fall within the cached interval [19]. Thus, some techniques that capture the change pattern of a sensor reading using data models such as the linear regression [2] and statistical distribution functions [9] have been proposed.

To estimate sensor readings, Tulone and Madden devised PAQ [18], which is based on the stationary time series model called an autoregressive model (AR). Particularly, in [18], a dynamic AR(3) model is used in which a future reading is predicted using recent three readings with the following equation:

(1) $$\begin{array}{c} \;X\left(t\right)=\alpha X\left(t-1\right)+\beta X\left(t-2\right)+\gamma X\left(t-3\right)+b\left(\omega \right)N\left(0,1\right), \end{array}$$

where *b*(*ω*)*N*(0,1) represents the Gaussian white noise of zero mean and standard deviation *b*(*ω*). In PAQ, to predict the future reading accurately, the proper coefficients *α*, *β*, and *γ* of AR(3) model are required. Thus, PAQ requires a long learning phase to build the stationary data model.

Jain et al. suggested *Dual Kalman Filter* [8] which is based on the Kalman filter. In addition, recently, Min and Chung proposed EDGES [10] based on a variant of the Kalman filter, that is, multimodel Kalman filter. In these approaches, each sensor estimates its readings independently with its own model. And the mirror model for each sensor is in the base station. In this aspect, PAQ, the Dual Kalman filter, and EDGES are similar. Unlike PAQ, the Dual Kalman filter, and EDGES, CMOS considers the spatial correlations such that the change patterns of sensor readings of the neighbor sensors are the same or similar. To utilize this correlation, in our work, sensors in WSN are grouped as a cluster and each sensor has several Kalman filters each of which serves a different purpose (see details in Section 4).

Like our approach, in EDGES, sensors are partitioned into clusters and an efficient clustering mechanism is suggested. To reduce the communication overhead, EDGES groups sensors whose data transmission patterns are similar. This clustering mechanism of EDGES cannot be applied to our work since, in our work, the spatially correlated readings of cluster members are not sent to the cluster header. In other words, as mentioned above, in EDGES, each sensor estimates its reading independently. Instead, our work utilizes spatial correlation to predict cluster members' readings. In addition, in EDGES, the node failure is not considered. Thus, we suggest a more robust clustering mechanism.

In [2], the snapshot query approach was introduced. In this work, nodes can coordinate with their neighbors and elect a small set of representative nodes among themselves. Representative nodes maintain the sensor readings of their neighbor nodes using the linear regression. Let *v*_*i*_ be a sensor reading of node *n*_*i*_, and let *v*_*j*_ be the sensor reading of node *n*_*j*_. The estimator ${\hat{v}}_{j}$ is derived using the linear regression as follows: ${\hat{v}}_{j}=\alpha v_{i}+\beta$. If $|v_{j}-{\hat{v}}_{j}|<\epsilon$ (i.e., error bound); the authors say that *n*_*i*_ represents *n*_*j*_. In the snapshot approach, the node that can represent many other nodes becomes a representative node. In order to maintain the representative nodes, the authors assume that each node knows the values of its neighbors. For this, sensors periodically broadcast their readings to their neighbors as heartbeat messages. It wastes lots of energy since each node should receive the data of its neighbors. Also, since a representative node does not know its nonrepresentative nodes' data values within an interval of the nonrepresentatives' periodic data sending, the error bound cannot be guaranteed.

Since representative nodes maintain the sensor readings of their neighbors, their energy will be depleted faster. However, in the work of [2], this issue is addressed: a representative node that wastes its energy invites other nodes or uses LEACH data routing protocol [20]. LEACH is one of the most frequently referenced methods that allow each cluster to reselect the cluster header at proper intervals. The basic assumption of LEACH and its variants is that all sensor nodes can communicate with each other directly. Thus, when the communication distance is restricted, LEACH cannot be applied.

## 3. Preliminary

In order to predict a future value, many methods such as the linear regression and the Bayesian network have been proposed. Among them, one of the most well-known and often used tools is the *Kalman Filter* [21] which is introduced by Kalman as a recursive data processing algorithm for the discrete-data linear filtering problem. The Kalman filter is used in diverse applications such as signal processing and pattern matching. Since the feature of the Kalman filter is well summarized in [8, 10], this section will provide an overview of the Kalman filter briefly. For more details refer to [22].

**Figure 1:**
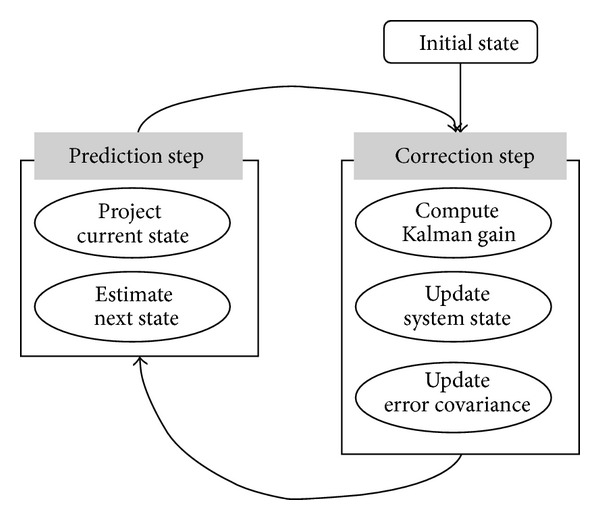
Recursive cycle of the Kalman filter.

The Kalman filter consists of mathematical equations that estimate the internal states of a system using a predictor-corrector type estimator as shown in Figure 1.

In the Kalman filter, the system model is represented by the following equations:

(2) $$\begin{array}{c} x_{k}=Fx_{k-1}+w_{k-1}, \end{array}$$

(3) $$\begin{array}{c} z_{k}=Hx_{k}+v_{k}. \end{array}$$

Equation (2) represents a process model that shows the transformation of the process state. Let *x*_*k*_ ∈ *ℜ* ^*n*^ be the state of a process. *F* is the *n* × *n* state transition matrix relating the state *x*_*k*_ and *x*_*k*−1_. Equation (3) represents a measurement model that describes the relationship between the process state and the measurement *z*_*k*_ ∈ *ℜ* ^*m*^. *H* is the *m* × *n* matrix relating the state to the measurement. *w*_*k*_ ∈ *ℜ* ^*n*^ and *v*_*k*_ ∈ *ℜ* ^*m*^ represent the process noise and measurement noise, respectively. The covariance for *w*_*k*_ and *v*_*k*_ are *Q* and *R*, respectively.

In order to estimate the process state *x*, the Kalman filter uses estimators $\hat{x_{k}}$ and $\hat{x_{\bar{k}}}$. $\hat{x_{k}}$ is called a *posteriori* state estimate at time *k* given measurement *z*_*k*_. And $\hat{x_{\bar{k}}}$ is called a *priori* state estimate at time *k* using a previously estimated posterior state $\hat{x_{k-1}}$. $\hat{x_{\bar{k}}}$ and $\hat{x_{k}}$ are computed by the following equations:

(4) $$\begin{array}{c} \hat{x_{\bar{k}}}=F\hat{x_{k-1}}, \end{array}$$

(5) $$\begin{array}{c} \hat{x_{k}}=\hat{x_{\bar{k}}}+K_{k}\left(z_{k}-H\hat{x_{\bar{k}}}\right). \end{array}$$

In the discrete Kalman filter, by using (4), the prediction of a future value is conducted. And, by using (5), the correction of an estimated value (i.e., measurement update) is performed.

In (5), the *n* × *m* matrix *K*_*k*_ is called *Kalman gain*. One form of the *K*_*k*_ is given by

(6) $$\begin{array}{c} K_{k}=P_{\bar{k}}H^{T}{\left(HP_{\bar{k}}H^{T}+R\right)}^{-1}=\frac{P_{\bar{k}}H^{T}}{HP_{\bar{k}}H^{T}+R}. \end{array}$$

In (6), $P_{\bar{k}}$ is the a priori estimate error covariance which is derived as follows:

(7) $$\begin{array}{c} P_{\bar{k}}=E\left[\left(x_{k}-\hat{x_{\bar{k}}}\right){\left(x_{k}-\hat{x_{\bar{k}}}\right)}^{T}\right]=FP_{k-1}F^{T}+R. \end{array}$$

The a posteriori estimate error covariance *P*_*k*_ is derived as follows:

(8) $$\begin{array}{c} P_{k}=E\left[\left(x_{k}-\hat{x_{k}}\right){\left(x_{k}-\hat{x_{k}}\right)}^{T}\right]=\left(I-K_{k}H\right)P_{\bar{k}}. \end{array}$$

As presented in the above equations, the Kalman filter does not store the previous data set nor reprocesses stored data if a new measurement becomes available. In other words, to predict a future value at time *k*, the Kalman filter only requires the previously predicted future value at time *k* − 1 and a measurement value at time *k* [10].

## 4. CMOS

In this section, we present our proposed technique, CMOS, that groups sensors into clusters and monitors sensor readings utilizing the spatial correlation.

It is widely accepted that the energy consumed for one bit transfer of data can be used to perform a large number of arithmetic operations in the sensor processor. Thus, we do not consider the computation cost in our work. We assume that each sensor has the same communication distance *c*.

### 4.1. Basic Idea of CMOS

A comprehensive study [23] on routing algorithms found that the cluster based routing algorithms are more energy efficient compared to the direct algorithms in which each sensor node directly transmits the sensor reading to the base station. Also, a direct algorithm requires that all sensor nodes send data directly to the base station, which contradicts the limited transmission capability of sensor nodes. Therefore, these algorithms cannot actually be used in many real applications.

Thus, in CMOS, sensor nodes in a network are grouped into clusters and each cluster elects a cluster header. A cluster header communicates with the base station through multihop routing. The maximum distance between a cluster header and its member nodes is *c* (i.e., one hop distance). Since member nodes and the respective cluster header are located closely, the spatial correlation such that the changing patterns of sensor readings of the neighbor sensors are the same or similar occurs.

**Figure 2:**
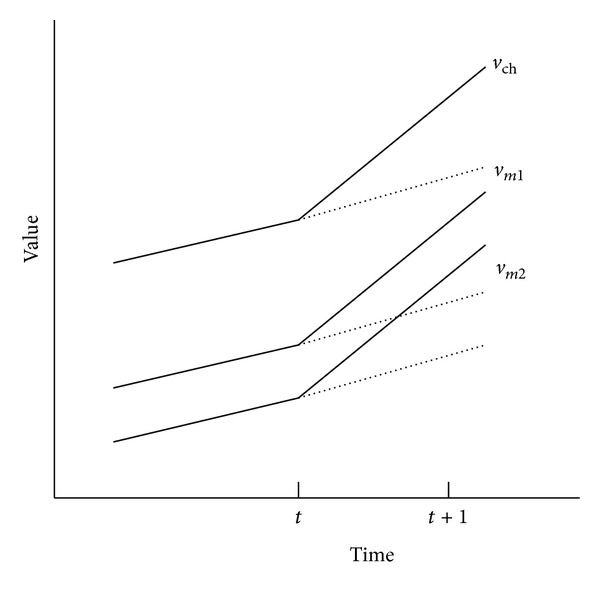
Simple situation.

Figure 2 illustrates the basic idea of our work. Suppose that a cluster header CH has two member nodes *m*1 and *m*2 within the communication range *c*.

In the previous techniques such as the Dual Kalman [8], EDGES [10], and PAQ [18], each sensor node keeps its own data model to predict its reading independently. As shown in Figure 2, sensor readings *v*_ch_, *v*_*m*1_, and *v*_*m*2_ are of CH, *m*1, and *m*2, respectively, change at time *t*. The dotted lines represent the estimated values. If the gaps of actual readings and estimated readings are greater than the user specific threshold *ϵ*, the member nodes *m*1 and *m*2 send their actual readings to CH. After CH collects the sensor readings of the members and then sends the collected readings and its reading to the base station. Thus, at least three messages are sent (i.e., two messages from *m*1 and *m*2 to CH and one message from CH to the base station).

In contrast to the previous techniques, in CMOS, a member node keeps a data model to maintain the difference of its reading and the cluster header's reading. As mentioned above, there is the spatial correlation on the neighbor nodes. Although sensor readings of sensors change at time *t* + 1, the difference of CH's reading and member's reading is stable due to the spatial correlation. By using this feature, we reduce the number of message sent.

In addition, CMOS exploits a basic but important property of WSNs; that is, a node broadcasts messages to its neighbors. In CMOS, CH broadcasts its reading *v*_ch_ to member nodes at time *t* + 1 due to the failure of prediction. Since the member nodes *m*1 and *m*2 maintain the data models to keep the differences of their readings and CH's reading, the member nodes identify the differences as stable although *v*_*m*1_ and *v*_*m*2_ change. Thus, *m*1 and *m*2 do not react to the broadcasting of CH. Then, CH can infer that the differences do not change and CH sends its readings to the base station. In this case, at least, two messages are sent (i.e., one broadcast to members and one message sent to the base station).

### 4.2. Behavior of CMOS

As mentioned earlier, CMOS estimates sensor readings using the Kalman filter. For the data model of the Kalman filter, we use the uniform velocity model since it is simple and hence it requires low computing cost. In CMOS, *x*_*k*_ = [*v*_*k*_, *r*_*k*_]^*T*^ is used as a process state where *v*_*k*_ is a value and *r*_*k*_ is the rate of change (i.e., velocity) of *v*_*k*_. Under the uniform velocity model, *v*_*k*_ = *v*_*k*−1_ + *r*_*k*−1_Δ*t* and *r*_*k*_ = *r*_*k*−1_, where Δ*t* is an elapse time between *k* and *k* − 1. Thus, we make a state transition matrix *F* as follows:

(9) $$\begin{array}{c} F=\left[\begin{array}{cc} 1 & \Delta t\\ 0 & 1 \end{array}\right]. \end{array}$$

Then, let the measurement of a value (i.e., the actual value) be *z*_*k*_ ∈ *ℜ*. The state measurement matrix *H* is represented as follows:

(10) $$\begin{array}{c} H=\left[\begin{array}{cc} 1 & 0 \end{array}\right]. \end{array}$$

In CMOS, a cluster header CH estimates its reading *v*_ch_ based on the process model and measurement model (i.e., *F* and *H*). If the difference of the actual value *v*_ch_ and the estimate value ${\hat{v}}_{\text{ch}}$ is greater than *ϵ* (i.e., $|v_{\text{ch}}-{\hat{v}}_{\text{ch}}|>\epsilon$), CH will report *v*_ch_ to the base station. Otherwise, the base station can obtain ${\hat{v}}_{\text{ch}}$ as a report value using the Kalman filter KF_CH_ for CH.

A member node *mi* maintains the difference *d*_*mi*_ between its reading *v*_*mi*_ and the cluster header's report value *v*_ch_rep__ (i.e., *d*_*mi*_ = *v*_*mi*_ − *v*_ch_rep__) using the Kalman filter KF_*d*_*mi*__ under the uniform velocity model. As mentioned above, the cluster header CH estimates *v*_ch_ as ${\hat{v}}_{\text{ch}}$. Thus, in a member node, the cluster header's report value *v*_ch_rep__ is ${\hat{v}}_{\text{ch}}$ if the cluster header does not broadcast *v*_ch_ (i.e., $\left|v_{\text{ch}}-{\hat{v}}_{\text{ch}}\right|\leq \epsilon$). Otherwise, *v*_ch_rep__ is *v*_ch_.

**Figure 3:**
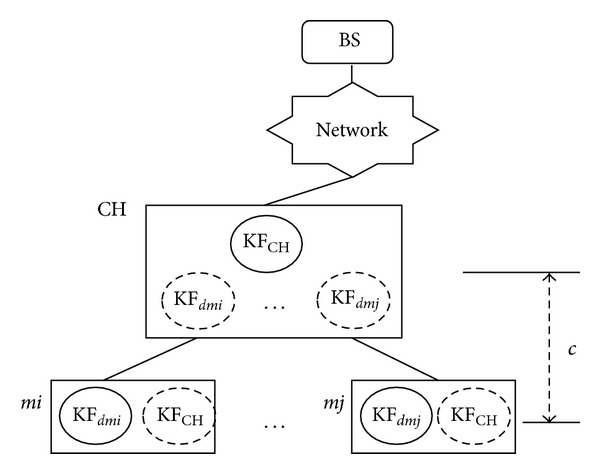
An architecture of a cluster.

The basic architecture of a cluster in CMOS is presented in Figure 3. As shown in Figure 3, CH has the Kalman filter KF_CH_ in order to estimate its reading *v*_ch_. Each member node has the mirror KF_CH_ represented as a dotted circle in Figure 3.

Each member node *mi* also has the Kalman filter KF_*d*_*mi*__ in order to estimate the difference *d*_*mi*_ of its own reading and CH's reading. CH has the mirror KF_*d*_*mi*__s for its member nodes. In addition, the base station keeps the information of the clusters including the Kalman filters for cluster headers and their members. Thus, the base station can estimate properly sensor readings which are measured in a cluster properly.

At a time *t*, if CH does not broadcast *v*_ch_, a member node can obtain $v_{\text{c}{\text{h}}_{\text{rep}}}(={\hat{v}}_{\text{ch}})$ using the mirror KF_CH_. Otherwise, a member node listens to *v*_ch_rep__( = *v*_ch_) and updates the mirror KF_CH_ (CH also updates KF_CH_).

Then, every member node *mi* computes ${\hat{d}}_{mi}$ using KF_*d*_*mi*__ and gets the sensor reading *v*_*mi*_ from the sensor module. Using *v*_ch_rep__ and *v*_*mi*_, *mi* can compute *d*_*mi*_.

If $|d_{mi}-{\hat{d}}_{mi}|>\epsilon$, *mi* sends *d*_*mi*_ and updates KF_*d*_*mi*__. Then, CH gets *d*_*mi*_ and updates the mirror KF_*d*_*mi*__. If $|d_{mi}-{\hat{d}}_{mi}|\leq \epsilon$, *mi* does not send *d*_*mi*_ to CH regardless of broadcasting of *v*_ch_ from CH. Then, CH can compute ${\hat{d}}_{mi}$ using the mirror KF_*d*_*mi*__.

Finally, based on the following lemma, the cluster header CH can obtain *v*_*mi*_ or can estimate ${\hat{v}}_{mi}$ accurately using *d*_*mi*_ or ${\hat{d}}_{mi}$.

**Lemma 1**

In a cluster, the cluster header CH obtains the sensor reading *v*_*mi*_ of its member *mi* within *ϵ* using *d*_*mi*_ or ${\hat{d}}_{mi}$.

**Proof**

By definition of *d*_*mi*_, *v*_*mi*_ = *v*_ch_rep__ + *d*_*mi*_. Since *mi* sends *d*_*mi*_ to CH when $|d_{mi}-{\hat{d}}_{mi}|>\epsilon$, CH can get the exact *v*_*mi*_ whether CH sent its reading *v*_ch_ or not.

$\left|v_{mi}-{\hat{v}}_{mi}|=|(v_{\text{c}{\text{h}}_{\text{rep}}}+d_{mi})-(v_{\text{c}{\text{h}}_{\text{rep}}}+{\hat{d}}_{mi})|=|d_{mi}-{\hat{d}}_{mi}\right|$. Therefore, CH can guarantee that ${\hat{v}}_{mi}(=v_{\text{c}{\text{h}}_{\text{rep}}}+{\hat{d}}_{mi})$ will be within *ϵ*.

Finally, each cluster header CH sends *v*_ch_ if $|v_{\text{ch}}-{\hat{v}}_{\text{ch}}|>\epsilon$ as well as the received *d*_*mi*_ to the base station. Then, the base station can get the accurate sensor readings of sensor nodes using Kalman filters or the received values.

### 4.3. Cluster Management in CMOS

The cluster headers perform data transmission to the base station on behalf of the other sensor nodes within their respective clusters. The idea of CMOS is to have the cluster headers bear the brunt of the energy consuming data transmission to the base station, thereby allowing the other sensor nodes in a cluster to transmit data only to their nearby cluster header and avoid having to transmit data unnecessarily to the more distant base station. However, since the load of data transmission is shifted to the cluster headers, they exhaust their energy faster than the member nodes. Thus, in this section, we present a simple but efficient cluster management technique.

The basic idea of our approach is that a sensor node having much energy acts as a cluster header. To do this, our cluster management technique consists of four steps: initialization, header election, adjustment, and finalization.

**Figure 4:**
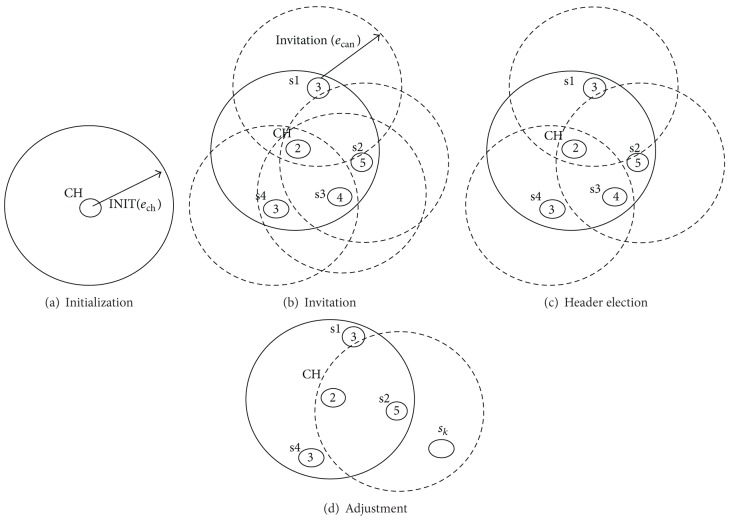
Cluster management steps.

Roughly speaking, in the initialization step, a cluster header that severely wastes its energy broadcasts its energy level in order to release its responsibility. In the header election step, some member nodes whose energy levels are greater than those of the current header become headers. Note that in this step, all member nodes having lower energy cannot be headers. In the adjustment step, new cluster headers form new clusters. Finally, we check that there are nodes that do not participate in new clusters in the finalization step.  Figure 4 illustrates our cluster management technique.

*Initialization.* In this step (see Figure 4(a)), a cluster header broadcasts an INIT message with its current energy level *e*_ch_ to its neighbors. The repetitive cluster management requires additional consumption of sensor energy. To avoid frequent cluster adjustment, an INIT message is broadcasted by a cluster header CH whose reduction ratio (= (Previous Energy – current Energy)/Previous Energy) is greater than a threshold ET, where Previous Energy denotes the energy level when CH started to act as the cluster header and current Energy denotes the current energy level of the cluster header. In addition, since the energy reduction ratio can exceed ET by a small number of data sent when an energy level becomes very low, we restrict CH from invoking the initialization step within a time interval TT after the previous broadcasting.

*Header Election*. Upon receiving an INIT message with the energy level *e*_ch_, member nodes whose energy levels are greater than *e*_ch_ become candidate headers. These candidate headers broadcast the INVITATION messages with their energy level *e*_can_ (see Figure 4(b)). Now, the member nodes will know about the candidate nodes within the communication distance *c*. A candidate node can be a new cluster header if one of the following conditions is satisfied: (1) there is no other candidate node within *c*, (2) there is no other candidate node having higher energy than itself, and (3) it cannot join another cluster although there is a candidate node having higher energy than itself. For example, as shown in Figures 4(b) and 4(c), suppose that the energy levels of CH, s1, s2, s3, and s4 are 2, 3, 5, 4, and 3, respectively. s1 becomes a new cluster header by condition (1). Also, s2 becomes a new cluster header by condition (2). In addition, s4 becomes a new cluster header by condition (3) because s3 will be a member of s2 although s3, whose energy level is 4, is in the communication range of s4.

*Adjustment*. Nodes except CH and new cluster headers broadcast JOIN messages to a new cluster header within the communication range. Then, a new cluster header forms a cluster using JOIN messages. Thus, a new cluster header replies ACK with respect to a JOIN message of a node. Then, a node receiving ACK notifies the current header of a change in headers. In this case, a node which was not a member of CH but received an INVITATION message with *e*_can_ (e.g., node *s*_*k*_ in Figure 4(d)) changes its header if the energy level of *s*_*k*_'s header is less than *e*_can_.

A member node knows the initial energy level of its header. Also, in order to avoid the collision of data sending via broadcasting media, a node overhears its neighbor's data transmission when it wakes. If a node overhears its header's data transmission, a node reduces the energy level of its header. Otherwise, the energy level of its header is not changed. Thus, each node estimates the energy level of its header. We guarantee that the actual energy level of its header is less than or equal to the estimated energy level.

*Finalization*. In the adjustment step, the previous cluster header CH did not participate. In this step, we decide whether CH plays its role again or not. As mentioned above, a cluster header knows its members. After the adjustment step, CH knows whether a member node changed its header to a new cluster header or not. Thus, if there are nodes which are not covered by new cluster headers, CH is reselected as a cluster header for these nodes. In contrast, if all member nodes of CH join new clusters, CH also participates in a new cluster as a member. Note that, during header election and adjustment phases, CH knows new cluster headers among its members and their energy level. Therefore, CH can choose its new cluster header easily.

As explained above, in our cluster management, each node makes an autonomous decision without any centralized mechanism. This feature allows us to make a robust system. There are two types of failures: link failure and node failure. By retransmission, link failure can be solved easily. For node failure, every node broadcasts a beacon signal periodically. Thus, a node can detect the failure of a neighbor node if a neighbor node does not send a beacon signal for a long interval. If a cluster header detects the failure of a member node, it excludes the failure member from its cluster. If member nodes detect the failure of their cluster header, they assume that there is an INIT message with an energy level 0. Then, the remaining three steps are performed.

## 5. Performance Study

In this section, we demonstrate the efficiency of our proposed method, CMOS. We perform simulations to compare the performance of CMOS with snapshot approach (SS) [2], PAQ [18], Dual Kalman filter (DKF) [8], and EDGES [10] on the synthetic data sets. In our experiments, we find that CMOS shows significantly better performance.

### 5.1. Simulation Setup

We begin with the description of the synthetic data set and parameters used in our experiments. The default parameter setting used in our experiments is summarized in Table 1. The sensor network consists of 100 and 500 sensors, randomly located in the [0, 100) × [0, 100) two-dimensional-sensing field.

According to the approximation techniques, some specific parameters such as outlier bound are required. In PAQ, the quality of a data model is determined by *δ*. If the prediction error falls outside [−*ϵ*, *ϵ*], the prediction model is reconstructed. Also, if the error falls within [−*ϵ*, −*δ*]∪[*δ*, *ϵ*], a node opens a buffer sized *B* (if it is not open). If the number of outliers exceeds *B*/2, the prediction model is reconstructed with the *B* sized buffer. In addition, a buffer is required to make a liner regression model in the snapshot technique. In our experiment, we set the size of the buffer for the snapshot technique as equal to that of PAQ. In the snapshot technique, every sensor broadcasts its value per an interval of 25 time units to maintain the linear regression model.

For the synthetic data, we make two data sets: Wave, and EnergyDisperse. For the Wave data set, we assign a value in the range [0.0,…, 50.0] to a location in the [0, 100) space using the SIN function. We set the values to the two-dimensional space using the assigned values, where locations with the same *x*-coordinates have the same value. Then, we simulate the wave passing as the vertical shift from left to right.

The EnergyDisperse data set is used to simulate the behavior of energy dispersion. For each time *t*, the value *V*(*i*, *j*)_*t*_ at a location (*i*, *j*) is changed according to the following equation:

(11) V(i,j)t=V(i,j)t−1 +α(V(i+1,j)t−1+V(i−1,j)t−1   +V(i,j+1)t−1+V(i,j−1)t−1−4V(i,j)t−1).

In the above equation, *α* is a dispersion factor. In this experiment, we set *α* to 0.25. By using the above equation, the state of the sensing field reaches the equilibrium state as time passes. Thus, we randomly select 10 locations and assign 50 to the values of the selected locations. Also, for every 100 time units, we change the selected locations randomly.

In addition, we locate the base station at the center of the sensing field for all data sets. The communication distance *c* on the synthetic data is 20.

### 5.2. Simulation Results

To measure the effect of our cluster management method, we make two versions of CMOS: CMOS_fix_ and CMOS_adj_. In CMOS_fix_, initial clusters are not changed. Thus, CMOS_fix_ shows the efficiency of our basic idea.

To measure the energy consumption in diverse environments, we use three error bounds, 0.2, 0.1, and 0.05. To obtain the energy consumption of each technique, we run the simulator for the interval of 3000 time units. To compute the energy consumption, we use the free space channel model [20]. Under this model, to transmit an *l*-bit message over a distance *c*, a sensor expends

(12) $$\begin{array}{c} E_{T}\left(l,c\right)=E_{T-\text{elec}}\left(l\right)+E_{T-\text{amp}}\left(l,c\right)=l*E_{\text{elec}}+\xi_{\text{amp}}*l*c^{2}. \end{array}$$

To receive this message, a sensor expends

(13) $$\begin{array}{c} E_{R}\left(l\right)=E_{R-\text{elec}}\left(l\right)=l*E_{\text{elec}}, \end{array}$$

where *E*_elec_ denotes the energy consumption for running the transmitter or receiver circuit and *ξ*_amp_ denotes the energy consumption for a transmit amplifier. In this experiment, we set 50 nJ/bit to the electronic circuit constant (*E*_elec_) and 100 pJ/bit/m^2^ to the transmit amplifier constant (*ξ*_amp_). Based on the above energy model, we implement our own simulator using JDK 1.6 and run on MS Windows 7.

**Figure 5:**
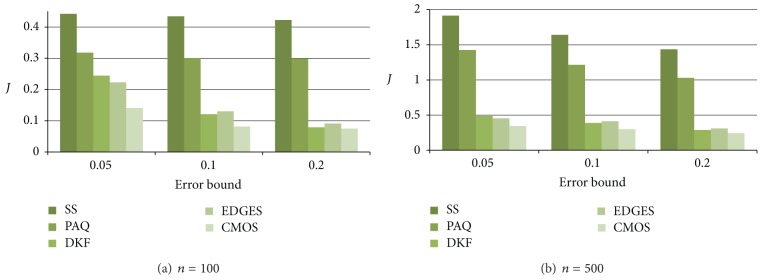
Average energy consumption on Wave data.

**Figure 6:**
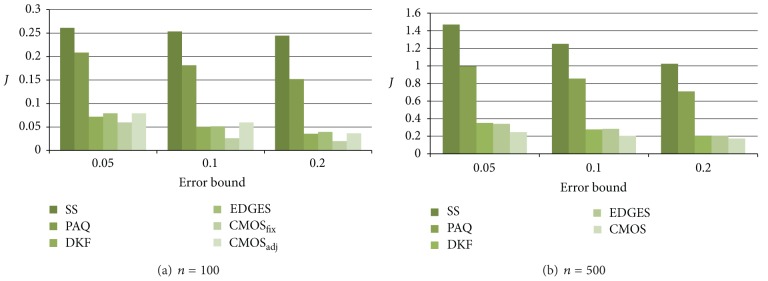
Average energy consumption on EnergyDisperse data.

Figures 5 and 6 show the energy consumption on the Wave data and the EnergyDisperse data, respectively, averaged over sensors. Generally, as the error bound increases, the energy consumption decreases since the number of data transmissions decreases.

The snapshot technique shows the worst performance due to periodic data broadcasting. In addition, since PAQ is based on the AR model, PAQ requires a long learning phase. When data is (weak) stationary, the data model used in PAQ can estimate the future value accurately. However, our experimental data sets reflecting real world are changed as time passes. Thus, PAQ shows the worst performance compared to DKF, EDGES, and CMOS.

As shown in Figures 5 and 6, DKF, EDGES, and CMOS_adj_ show similar performances. CMOS_fix_ shows the best performance over all cases. This result indicates that our approximate data monitoring technique based on spatial correlation presented in Section 4.2 is effective over all cases. In addition, as shown in Figures 5(a) and 5(b) as well as Figures 6(a) and 6(b), when the number of sensor nodes increases, the performance gap between PAQ and CMOS_fix_ increases. This results show that CMOS is more scalable than PAQ.

The experimental result of CMOS_adj_ shows the performance of the combination of our data monitoring technique and cluster management technique. As we expected, CMOS_adj_ shows a slightly worse performance compared to CMOS_fix_ since there is additional communication overhead in order to maintain clusters autonomously as described in Section 4.3.

Furthermore, as presented earlier, since the SIN wave gradually moves as time passes on Wave data, the data change patterns between neighbors are similar. In contrast, on EnergyDisperse data, some sensor readings suddenly change since we randomly assign the highest value to some sensors. Therefore, the performance of CMOS_adj_ is better than that of EDGES on Wave data as shown in Figure 5 since our work is based on the spatial correlation but, in EDGES, each sensor estimates its reading independently. In other words, to estimate a sensor reading, EDGES utilizes temporal correlation only. However, on some cases of EnergyDisperse data, CMOS_adj_ shows worse performance compared to EDGES as shown in Figure 6 since EDGES uses more complicate Kalman filter (i.e., multi-modal Kalman filter) compared to CMOS_adj_.

**Figure 7:**
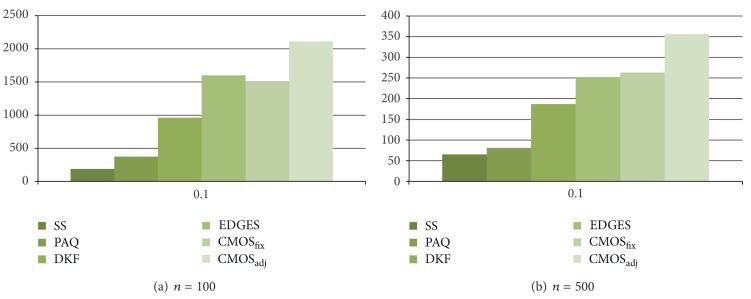
Time to a sensor failure on Wave data.

**Figure 8:**
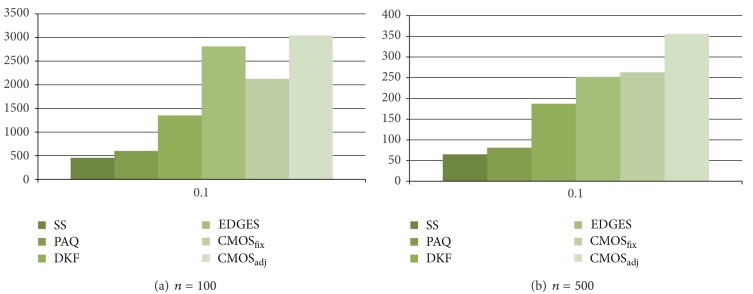
Time to a sensor failure on EnergyDisperse data.

As presented in the performance results for energy consumption in Figures 5 and 6, CMOS_adj_ and EDGES are worse than CMOS_fix_ due to the cluster management overhead. To show the effectiveness of our cluster management method, we restrict the initial energy of a sensor to 1 J and measure the time that a sensor in the network drains its whole energy when *ϵ* = 0.1. Figures 7 and 8 show the experimental results for the lifetime.

As shown in Figures 7 and 8, CMOS_adj_ shows the best effectiveness over most of all cases. Like our work, EDGES also maintains clusters dynamically. However, as shown in Figure 7(b), the performance of EDGES is little bit worse than that of CMOS_fix_ on Wave data. This result indicates that our work utilizing spatial correlation efficiently estimates sensor reading. But, on EnergyDisperse data, the performances of EDGES and CMOS_adj_ are similar. On the average, CMOS_adj_ extends the lifetime of a network about 16.8%, 40.1%, and 122% compared to EDGES, CMOS_fix_, and DKF, respectively. This result indicates that our cluster management technique, with consideration to the energy level, enlarges the network lifetime although the average energy consumption of CMOS_adj_ is greater than that of a fixed cluster approach.

## 6. Conclusion

WSN has gained increasing importance due to its potential benefits for some civil and military applications such as combat field surveillance, security, and disaster management.

In this paper, we propose an efficient cluster based monitoring technique called CMOS. In CMOS, sensors in networks are grouped into clusters. The cluster header in a cluster predicts its reading and member nodes predict the differences of their readings and the cluster header's reading using the Kalman filters. Since each node keeps the mirror Kalman filter for the counterpart, a cluster header (member) node can estimate the reading of a member (header) without data transmission.

Each sensor node can reduce the amount of data transmitted due to the physical proximity to the cluster header. Unfortunately, these transmission loads are shifted to the cluster header. This unbalanced energy consumption of the CH can quickly disable the entire network. Thus, we propose an effective cluster management scheme to prolong the lifetime of sensor networks. Since in our cluster management technique each sensor makes a decision autonomously, the network is robust.

To show the efficiency of CMOS, we conduct an experimental study with synthetic data sets. The experimental results show that applying the spatial correlation reduces the energy consumption of each sensor and applying our cluster management technique extends the lifetime of a sensor network with small additional energy cost.

## Figures and Tables

**Table 1:** Parameters.

Parameter	Default value	Comments
Number of nodes (*n*)	100 or 500	
Communication distance (*c*)	20 or 10	
Error bound (*ϵ*)	0.1	
Energy threshold (EE)	30%	For cluster management
Time threshold (TT)	100	For cluster management
Outlier bound (*δ*)	*ϵ*/2	For PAQ
Buffer size (*B*)	25	For PAQ and snapshot
